# Adolescent over-general memory, life events and mental health outcomes: Findings from a UK cohort study

**DOI:** 10.1080/09658211.2015.1008014

**Published:** 2015-02-26

**Authors:** Catherine Crane, Jon Heron, David Gunnell, Glyn Lewis, Jonathan Evans, J. Mark G. Williams

**Affiliations:** ^a^ Department of Psychiatry, University of Oxford, Oxford, UK; ^b^ School of Social and Community Medicine, University of Bristol, Bristol, UK; ^c^ Mental Health Sciences Unit, University College London, London, UK

**Keywords:** Avon Longitudinal Study of Parents and Children, Adolescence, Depression, Suicidality, Autobiographical memory

## Abstract

Previous research suggesting that over-general memory (OGM) may moderate the effect of life events on depressive symptoms and suicidality has sampled older adolescents or adults, or younger adolescents in high-risk populations, and has been conducted over relatively short follow-up periods. The authors examined the relationship between OGM at age 13 and life events and mental health outcomes (depression, self-harm, suicidal ideation and planning) at age 16 years within a sample of 5792 adolescents participating in the Avon Longitudinal Study of Parents and Children (ALSPAC), approximately 3800 of whom had also provided data on depression and self-harm. There was no clear evidence of either direct or interactive effects of OGM at age 13 on levels of depression at age 16. Similarly there was no clear evidence of either direct or interactive effects of OGM on suicidal ideation and self-harm. Although there was some evidence that over-general autobiographical memory was associated with reduced risk of suicidal planning and increased risk of self-harm, these associations were absent when confounding variables were taken into account. The findings imply that although OGM is a marker of vulnerability to depression and related psychopathology in high-risk groups, this cannot be assumed to generalise to whole populations.

For many years we have been interested in the impact of the way people recall personal events from their past on future psychopathology. Using the Galton–Crowitz cue-word paradigm, Williams and Broadbent (1986) reported that suicidal patients showed a strong tendency to retrieve autobiographical memories in an over-general way, failing to retrieve one particular episode (Williams & Broadbent, 1986). For example, to the cue-word “enjoy” they gave responses such as “I used to enjoy a good party”, without mentioning a particular episode, rather than responding with “Jane’s birthday party last year”. These early findings with suicidal patients have been replicated, and further studies have shown that over-general recall is highly characteristic of patients with a diagnosis of major depressive disorder (MDD) and post-traumatic stress disorder (PTSD; see Moore & Zoellner, 2007; Sumner, Griffith, & Mineka, 2010; van Vreeswjik & de Wilde, 2004; Williams et al., 2007, for reviews).

Interest in over-general memory (OGM) and its mechanisms has been maintained in part because of research suggesting it is predictive of more persistent depressive symptoms. The possibility that OGM predicts downstream depressive symptoms (that is, that the causal direction is from a cognitive precursor to an affective outcome) has aroused interest among clinicians who are required to search for long-term vulnerability factors in their patients in order to ensure that, following intervention, they do not still have any factors that would increase the risk of relapse. OGM seems to be such just a vulnerability factor. Greater levels of OGM within a depressive episode have been shown in several studies to predict level of depressive symptoms at follow-up, even after controlling for baseline symptom severity (Brittlebank, Scott, Williams, & Ferrier, 1933; Dalgleish, Spinks, Yiend, & Kuyken, 2001; see Sumner et al., 2010, for meta-analysis). Further, experimental induction of OGM in the laboratory has shown that it has a causal influence on psychopathology (Williams et al., 2006). This article is concerned with OGM as a vulnerability factor in adolescence. Before addressing these questions in more detail, we first give some defining characteristics of OGM, then some theoretical background to the mechanisms underlying the phenomenon.

Although early studies referred to *any* memory that was not specific as “over general”, the nature of the memory deficit was further elucidated by distinguishing different types of OGMs. Williams and Dritschel (1992) differentiated between memories that were over general by virtue of referring to a whole class of events, so-called categoric memories (e.g., “all the times I’ve failed exams”), and memories which were over general because they referred to an extended period of time, so-called extended memories (e.g., “my first semester at University”). They showed that group differences between depressed patients and controls were wholly due to increased retrieval of categoric memories while there were no group differences in number of extended memories. Despite this clarification, OGM has come to mean any response that fails to elicit a specific event (something that happened on a specific day).

Williams et al. (2007) developed the CaR-FA-X model, suggesting that in voluntary (top-down) search through the memory hierarchy (Conway & Pleydell-Pearce, 2000), variation in three mechanisms explained over-general recall. The first mechanism (CaR) refers to capture and rumination—in which self-referent material produced during the early stage of the retrieval process diverts the search for an episodic memory. For example, to the cue word, *happy*, a person may react with the thought “Why can’t I be as happy as others seem to be” and having been “captured” by this thought, starts to ruminate. The retrieved output is thus an OGM related to the rumination (“I was happy whenever I went out with Ben”). The second mechanism is functional avoidance (FA)—the tendency to passively avoid any episodic material (even positive material) that might reactivate memory of past difficulties or trauma. The third mechanism (X) refers to e*x*ecutive control or capacity limitations, given the effortful nature of top-down search. Each of these mechanisms has received support in the literature (for a review, see Sumner, 2012).

Most of the research has examined the relations between OGM and emotional pathology (depression, PTSD). Here, we are interested in the association between OGM and both depression and suicidal ideation/behaviour. But what sort of association might we expect to see? A meta-analysis of studies looking at the prospective effect of OGM on depressive symptoms, conducted by Sumner et al. (2010), found that such associations were significantly stronger for participants *who were depressed at the time of autobiographical memory assessment*, relative to those who were not (i.e., depression status moderated the relationship between OGM and depressive symptoms at follow-up). Sumner et al.’s data thus questioned the role of OGM as a predisposing vulnerability factor in the absence of pre-existing depression.

Two recent studies have extended this work to explore the association between OGM and initial onset of depression in high-risk (non-depressed, but depression-vulnerable) adolescents. The first, Hipwell, Sapotichne, Klostermann, Battista, and Keenan (2011), found an association between OGM to positive cue words and depressive symptoms at one-year follow-up in a high-risk sample of 11–12-year-old girls. The second, Rawal and Rice (2012), identified an association between OGM to negative cue words in high-risk adolescents with a family history of depression and onset of a first depressive episode in the following 12 months. These studies suggest that in some samples, OGM may indeed precede and predict the first onset of a depressive illness. However since both studies used high-risk samples, results must be interpreted with caution as it is not clear that differences in OGM in these studies were not themselves due to prior life events or prior depression. Further, evidence from broader community samples of adolescents is lacking, with several studies of non-clinical populations actually suggesting that OGM may be protective against distress in certain circumstances (e.g., Hermans et al., 2008; Raes, Hermans, Williams, & Eelen, 2006).

In parallel to work examining direct associations between OGM and onset of depressive symptoms, several studies have examined the interaction between OGM and life stress in determining level of depressive symptoms (or onset of MDD) in adolescence or early adulthood (Anderson, Goddard, & Powell., 2010; Gibbs & Rude, 2004; Hamlat et al., 2014; Stange, Hamlat, Hamilton, Abramson, & Alloy, 2013; Sumner, Griffith, & Mineka, 2011). In each case these studies have shown evidence that the association between life events and depression was stronger for individuals with more pronounced OGM (i.e., there was a significant interaction effect), after controlling for baseline symptom severity (although not always diagnostic history of depression). These studies suggest that OGM in adolescence or young adulthood may increase subsequent vulnerability to depressive symptoms in the context of life stress. However since all existing studies have been based on relatively short follow-up periods, it is not clear how persistent such effects might be.

Prospective work examining associations between OGM and suicidal ideation and self-harm is much more limited than work examining depression. Self-harm is common in adolescence—with a lifetime prevalence of nearly one in five for a population of 16–17-year olds with higher rates in females (25.6%) than males (9.1%; Kidger, Heron, Lewis, Evans, & Gunnell, 2012). Despite this, it frequently does not receive medical attention (Hawton, Rodman, Evans, & Weatherall, 2002). Several studies have shown evidence of OGM in suicidal patients (Kaviani, Rahimi-Darabad, & Naghavi, 2005; Pollock & Williams, 2001; Williams & Broadbent, 1986) and interestingly, although individuals with suicidal ideation typically have high levels of comorbid depression, this does not seem to entirely account for the levels of OGM observed in these populations (Leibetseder, Rohrer, Mackinger, & Fartacek, 2006). The rationale behind examining the association between OGM and suicidal behaviour has been the idea that OGM prevents adequate problem-solving, especially under stress, thus increasing the sense of hopelessness and inescapability that is known to underlie much suicidal behaviour. Specifically, Williams, Barnhofer, Crane, and Beck (2005) showed that OGM moderates the impact of mood on problem-solving for those with a suicidal history, but not for those with a similar history of depression but no suicidal history. However no studies have specifically examined associations between OGM and severity of suicidal ideation (for example, whether it included suicidal planning). Similarly no studies have examined associations between OGM and self-harm, outside particular high-risk patient groups such as those with borderline personality disorder (Startup et al., 2001) or a history of hospital-treated self-harm (e.g., Sinclair, Crane, Hawton, & Williams, 2007). It is therefore important to consider associations between OGM and self-harm in community samples of adolescents in addition to research in more restricted clinical populations.

The current study explored these issues, drawing on data collected from the Avon Longitudinal Study of Parents and Children (ALSPAC) birth cohort (Boyd et al., 2012; Fraser et al., 2012). A cohort study brings with it some disadvantages: the need to include relatively brief measures of each variable of interest and to administer some measures by self-completion questionnaire. However, it also has several strengths: access to a large and broadly representative community sample, the possibility of long-term follow-up and the ability to examine and control for a large number of sociodemographic confounding variables, which are not typically adequately assessed or controlled for in experimental or clinical studies. In the current study the autobiographical memory test (AMT) was included in a postal questionnaire sent to ALSPAC participants at age 13 (Heron et al., 2012). At age 16 years, participants reported on the occurrence and impact of a range of negative life events as well as reporting on several mental health outcomes: depressive symptoms, self-harm, suicidal ideation and suicidal planning. Because there is limited and inconsistent evidence concerning the associations between OGM and subsequent mental health in non-clinical community samples (with some evidence of the protective effect of over generality in community samples early on in the development of psychopathology; see Raes et al., 2006; Williams et al., 2007, for discussion), we did not have a strong hypothesis concerning the direction of association between OGM and later depression, suicidal ideation and self-harm for this population sample. However there is more consistent evidence that where an individual is exposed to negative life events or hassles, OGM may increase the impact of these events on mood (e.g., Hamlat et al., 2014). Thus we tested the hypothesis that OGM moderates the impact of life events on mental health outcomes, that is, increases the probability that a life event will result in depression or suicidality.

## METHOD

### Study population

The sample comprised participants from the ALSPAC (Boyd et al., 2012; Fraser et al., 2012). ALSPAC is an ongoing population-based study investigating a wide range of environmental and other influences on the health and development of children. Pregnant women resident in the former Avon Health Authority (Bristol) in south-west England, having an estimated date of delivery between 1 April 1991 and 31 December 1992 were invited to take part, resulting in a “core” cohort of 14,541 pregnancies and 13,973 singletons/twins alive at 12 months of age. Children enrolled in ALSPAC were more educated at 16 compared to the national average and less likely to be eligible for free school meals (an indicator of low income in the UK). Detailed information about ALSPAC is available via the study website (http://www.bris.ac.uk/alspac) which also contains a fully searchable data dictionary (http://www.bristol.ac.uk/alspac/researchers/data-access/data-dictionary). All aspects of the study are reviewed and approved by the ALSPAC Law and Ethics Committee, which is registered as an Institutional Review Board. Approval was also obtained from the Local Research Ethics Committees, which are governed by the Department of Health.

The primary source of data collection was via self-completion questionnaires administered at four points during the prenatal period then at regular intervals following birth to both parents and the “study child”. Since the age of 7 years the whole cohort has been invited to a regular “focus” clinic held on the ALSPAC premises for a variety of hands-on assessments.

### Predictors

##### AMT (adapted from Williams & Broadbent, 1986)

Autobiographical memory was assessed using 10 questions which were included in a large self-report postal questionnaire called “Food and Things” which was administered at age 13 and covered a wide range of topics. Ninety-five per cent of respondents were aged between 13 years 1 month and 13 years 3 months. Five positive (excited, happy, lucky, relaxed, relieved) and five negative (bored, failure, hopeless, lonely, sad) cue words which were familiar and relevant to young teenagers were selected from among word sets used in previous studies with adult samples and presented in pseudo-random order. Positive and negative cue words were matched for their frequency (and thus likely familiarity) in written material available for children using data from the University of Essex Children’s Printed Word Database (Lovejoy, 2003; see www.essex.ac.uk/psychology/cpwd). Although the AMT is often administered in an oral format, research shows good consistency between written and oral versions of the AMT, even with a significant time interval between testing sessions *(r* = .65, *n* = 90, *p* < .001; Raes et al., 2006), supporting the use of a written version as employed here. Raes and colleagues have used such a written version with children between 9 and 13 years old (both supervised and unsupervised with no timing restrictions) and found reliable and valid results (Raes, Verstraetan, Bijttebier, Vasey, & Dalgleish, 2010).

AMT instructions were shortened and simplified from those typically used with adults and no time constraint was imposed since the questionnaire was not administered under controlled conditions. Adolescents were asked to describe a “real event” that happened to them. Instructions stated that a response such as “I had a good time at Jane’s party” (a specific memory) would represent such an event whereas a response such as “I always enjoy a good party” (categoric memory) would not. This use of relatively minimal instructions is supported by the findings of recent research which suggests that such instructions provide a more sensitive test of memory specificity in non-clinical samples (e.g., Debeer, Hermans, & Raes, 2009). 

Responses were coded as specific, extended, categoric, associate or omission, following normal conventions, by one of two data preparation assistants, who were blind to any other characteristics of the young people. Ten per cent samples were double-rated at regular intervals, yielding an overall weighted *κ* of .82 (excellent agreement). Further details of the coding procedure can be found in Heron et al. (2012; Appendix B). The psychometric properties of the measure in this sample have also been investigated, indicating that there was no evidence of different dimensions based on cue valence, that is, all 10 responses lay on the same unidimensional continuum (see Heron et al., 2012). Given this, our planned analysis did not examine cue valence, as any differences found between different items would be likely to be chance findings or be due to unexplained item variance not attributable to the common variance shared by the items. The primary outcomes indicated the number of specific memory responses given, with extended, categoric, associate and omission responses falling into a single “non-specific” category. This measure was divided into quartiles for preliminary analysis but then used as a continuous measure, with a higher score indicating more specific memories, in regression models.

##### Life events measures (adapted from Barnett, Hanna, & Parker, 1983; Brown & Harris, 1978; Coddington, 1972)

As part of the postal questionnaire completed by young people when they were 16 years and 8 months (SD = 2.9 months—hereafter referred to as 16 years), they were asked to indicate whether or not they had experienced any of 20 life events at any time in the period since they were 12 years old. The events ranged from those that were likely to be severely negative (a close friend dying; a parent or sibling dying), to those that were likely to be disruptive but potentially neutral or positive (a parent’s new partner moving in, becoming a parent). Two unambiguously positive events—getting special recognition for good school work and getting a special prize or recognition for doing well in activities at school—were excluded, as was the event “becoming pregnant” since it was relevant to girls only.

**Figure 1.**
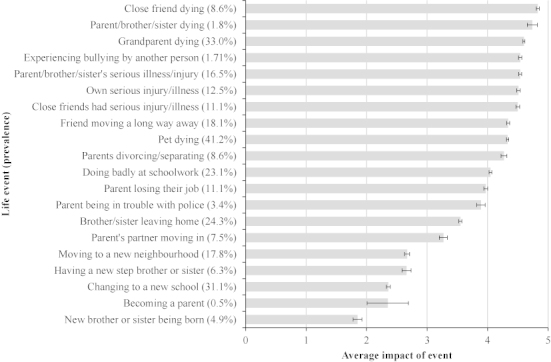
Frequency (in brackets) and average impact of life events occuring between 12 and 16 years, as reported by study participants at age 16.

For each event the young person indicated whether or not they had experienced the event and then rated the event impact on a scale ranging from 1—very pleasant to 5—very unpleasant. The reported impact data were used to derive a weighting for that event, for instance, among the 8.8% subgroup reporting that a close friend had died in the preceding 4.5 years the average impact rating was 4.8, this value was then used as a weighting for this event. Average impact ratings and rates of reporting of each life event are shown in Figure 1. Individuals were ascribed a weighted total score based on the particular events they reported. Using average weights rather than individual reported impacts was to reduce the effect of current psychopathology on the ratings of previous events. Although it might be argued that such a weighting procedure loses the individualised impact of any one event, it protects against the danger that short-term impact for any participant of, say, a pet dying, might get the same rating as a parent dying. The life events scale ranged from 0 to 73.1 with a median of 10.7. For preliminary analysis and to investigate the possibility of non-linear effects on the outcomes studied, the variable was divided into quartiles. For final regression models, a continuous standardised variable was used.

### Outcome measures

Four mental health outcomes were derived from additional responses to questions in the postal questionnaire at 16 years.

##### Suicidal ideation and self-harm

A series of questions related to the topic of self-harm and suicidal ideation, as previously described in Kidger et al. (2012). The “self harm” measure used here was defined to be a positive response to the question “have you ever hurt yourself on purpose in any way (e.g., by taking an overdose of pills or by cutting yourself)”? Similarly, “suicidal thoughts” were a positive response to “Have you ever thought of killing yourself, even if you would not really do it”? and “suicidal planning” a positive response to the question “Have you ever made plans to kill yourself”? These were chosen as similar questions have been used successfully in a number of previous population-based studies of adolescents (Hawton et al., 2002; Madge et al., 2008; Moran et al., 2012). Note that this measure does not distinguish between self-harming behaviour with or without explicit suicidal intent. The attribution of suicide intent is a matter of considerable current debate, especially in the light of the fact that some self-harming behaviour that has been previously seen as non-suicidal (such as self-cutting) may in fact predict later suicide (Bergen et al., 2012). This study assumes that all self-harm is potentially serious.

##### Depressive symptoms

The young people completed the 13-item short form of the Moods and Feelings Questionnaire (MFQ; Angold et al., 1995; Costello & Angold, 1988). This measure comprises a series of ordinal items measuring negative mood. The internal construct validity of a single continuum of severity of depressive symptoms has been supported in a UK community sample in which the items were subjected to unidimensional item-response modelling after simple binary recoding (Sharp, Goodyer, & Croudace, 2006). Cronbach’s *α* for these 13 items in the current data-set was found to be .91. For the purposes of these analyses, a binary measure of depressive symptoms was derived using the cut-off point of 10/11. This cut-off has been shown to have high sensitivity and specificity (Thapar & McGuffin, 1998) and has been applied in previous studies based on community samples (Angold, Erkanli, Silberg, Eaves, & Costello, 2002; Patton et al., 2008). This measure was dichotomised so that all outcomes would be binary and hence all models of the same form. The aim was to avoid the situation where moderating effects were being tested in both additive and multiplicative models since this was deemed detrimental to a clear interpretation. In this instance, conclusions were the same when using the MFQ in its continuous form (results available from authors).

### Confounders

##### Sociodemographic measures

Sociodemographic data were gathered from mothers through a questionnaire typically administered close to the point of entry to the study (i.e., during pregnancy or shortly after the study child’s birth). In the current analyses we adjusted for child gender, maternal marital status at delivery (never been married/remainder), parity (first born/second born/third born or greater), housing tenure (mortgaged or owned/privately rented/subsidised rented from council or housing association), overcrowding (more than one person per room/remainder), maternal education (no high school qualifications/high school/beyond high school), household income (quintiles of household disposable income when the child was a toddler and accounting for family size and composition, estimated housing benefits) and finally parental social class (the highest social class of either parent) at enrolment based on the Registrar General’s classification of occupations, I/II (professional/managerial and technical) versus IIINM or lower (skilled non-manual/manual, semiskilled and unskilled).

##### The role of depression at baseline

Information on depressed mood around the time of the completion of the AMT questions was obtained via an earlier administration of the MFQ. Adolescents completed the MFQ during a focus clinic held when they were approximately 12 years 10 months (interquartile range: 12 years 8 months to 12 years 11 months). *Exclusion*: a score of 11 on the MFQ scale was taken as our threshold for indicating the presence of possible depression and used as an exclusion criterion in a sensitivity analysis, with the purpose of assessing whether the results may have been influenced by the inclusion of participants with depressive symptoms around the time of AMT testing. *Adjustment*: regression models were also adjusted for the continuous MFQ scale which we refer to as depressed mood.

### Statistical methods

Analysis consisted of a series of univariable and multivariable logistic regression models, focusing on each binary outcome in turn. Estimates were adjusted for the potential confounding effects of the variables listed above prior to a final step in which the role of depressed mood at baseline was investigated through exclusion and adjustment. For the interaction models examining the potential moderating effect of AMT, regression models were derived containing weighted life events, continuous AMT and their product. We theorised that the positive association between life events and later psychopathology would attenuate in a linear fashion as memory specificity increased. In other words, one would expect the odds ratio for the product (interaction) term to be below one.

### Missing data considerations

There were three types of missing data to address for the proposed analyses. We discuss each in turn along with our remedy.

##### Partial response to the 10 AMT questions

Whilst 5792 participants completed at least some of the 10 AMT questions, only 1276 (22.0%) provided 10 usable text responses (Heron et al., 2012). Written instructions which accompanied the questions stated that if a memory could not be recollected for a particular cue word then the participant should just leave the line blank, and hence, in line with Griffith et al. (2009), these non-responses were assigned a score of zero. Response rates were lowest for items for which the young people were likely to have least experience of (e.g., hopelessness and failure) hence we did not feel that the observed (non-missing) AMT data were necessarily representative of a potential full set of responses. Given this, our chosen strategy of setting these missing values to zero was deemed preferable to either rescaling based on the number of actual responses or using imputation, which would both be making assumptions that were harder to justify.

##### Other incomplete data

Within the sample of 5792 with AMT information, information on life events, outcome measures and confounders was incomplete. To rectify this problem we employed the multivariate imputation by chained equations approach (van Buuren, Boshuizen, & Knook, 1999) using the “mi ice” routine (Royston, 2009) in Stata version 13.1 MP. The method is based on the missing at random assumption that, conditional on the other data in the imputation model, there should not be systematic differences between observed and missing values for a given variable. Imputation was used to estimate missing information among the sample of 5792 who had been assigned an AMT score. Auxiliary variables, strongly predictive of dropout and/or the variables suffering from missing data were used to assist with the imputation. One hundred imputed data-sets were produced, each with 20 cycles of regression switching.

##### Dealing with those providing sparse information

As a further sensitivity analysis we added an inverse probability weighting (IPW) to the analyses performed on the imputed data. The motivation for this additional step was that a substantial proportion of the ALSPAC cohort lacked an AMT rating. The IPW/multiple imputation approach has recently been proposed (Seaman, White, Copas, & Li, 2012) for the situation where one might not feel confident using imputation throughout the whole sample, e.g., due to areas of sparse data, or in our case due to the lack of strong auxiliary information which might be used to imputed missing AMT. For the IPW analysis, a logistic regression model was derived using data collected prior to age 13 to predict the availability of AMT data amongst the 10,434 who were sent the questionnaire containing the AMT questions. Predicted probabilities from this model were then used to weight the regression analyses to adjust for any bias due to systematic differences in those providing AMT information. Since this analysis had little impact on the regression estimates, these data are not shown but are available from the authors.

## RESULTS

### Socioeconomic and demographic characteristics (SES) and data availability

As stated previously, of the starting sample of 13,976, a total of 5792 (41.4%) participants provided data to enable an AMT score to be derived. Of the 8184 non-responders, 4642 were sent the questionnaire but did not respond and 3542 (25.3%) were not sent the questionnaire containing the AMT questions. The 5792 are the focus for these analyses. Of this subgroup, 3708 (64.0%) returned the later questionnaire at age 16 and provided information on current mood, self-harming and suicidal ideation. Table 1 shows the demographic characteristics of this sample providing predictor and outcome information (Column 6) compared to those for whom we have no AMT data, as well as those who completed the AMT questionnaire but provided no age 16 outcome data. Across a range of SES indicators those who had completed data at age 16 were more advantaged than those without such data. Additionally females were over-represented in the sample at 16 years compared to males.

**TABLE 1:** The association between demographics variables and data availability

			Data availability			
		*n*	No AMT data (*n* = 8184) (%)	AMT data but no 16 year response (*n* = 2084) (%)	AMT data plus 16 year response (*n* = 3708) (%)	*X^2^, p*
Gender	Female	6756	42.3	48.6	61.6	*X*^2^ = 383.0, *p* < .001
	Male	7220	57.7	51.4	38.4	
Housing tenure	Mortgaged/owned	9559	65.8	78.3	86.3	*X*^2^ = 589.6, *p* < .001
	Private rented	1384	12.5	9.2	7.5	
	Subsidised rented	2082	21.7	12.5	6.2	
Parity	First born	5770	42.0	45.0	49.8	*X*^2^ = 99.8, *p* < .001
	Second born	4539	35.1	36.2	34.5	
	Third born plus	2618	22.9	18.8	15.7	
Home overcrowding	≤1 person/room	11,924	90.8	94.8	96.9	*X*^2^ = 148.3, *p* < .001
	>1 person/room	878	9.2	5.2	3.2	
Maternal education	A level or higher	4392	28.1	34.7	49.4	*X*^2^ = 661.6 *p* < .001
	O level	4296	34.1	37.8	33.7	
	<O level	3728	37.8	27.5	16.9	
Household income	Top 20%	2010	16.9	17.7	26.2	*X*^2^ = 278.7, *p* < .001
	Middle 60%	5937	57.3	62.8	61.5	
	Lowest 20%	1992	25.8	19.5	12.4	
Social class	Professional/manager and technical	6339	48.5	56.1	66.2	*X*^2^ = 281.9, *p* < .001
	Skilled non-manual or lower	5162	51.5	43.9	33.8	
Marital status at enrolment	Married (+divorced/widow)	10,586	76.8	83.3	87.9	*X*^2^ = 201.0, *p* < .001
	Unmarried	2499	23.2	16.7	12.1	
Maternal age at delivery	<25 years	3337	30.1	19.1	12.9	*X*^2^ = 583.6, *p* < .001
	25–29	5403	38.7	41.2	37.2	
	30–34	3850	23.0	29.9	36.3	
	35+	1386	8.3	9.9	13.6	

### SES, AMT score and life events

We used the AMT data collected at age 13 to examine the effect of SES across quartiles of AMT across the same range of sociodemographic indicators (see Web Table 1). Males were more likely to score in the bottom quartile (low specificity) compared to females. Those in the bottom quartile were also more likely to live in subsidised rented accommodation, to live in overcrowded accommodation, to have a lower level of maternal educational qualifications, social class and household income, and to be born to a younger, unmarried mother. We also looked at the relationship between SES and life events, and as expected those in lower SES groups had been exposed to a greater number of life events (see Web Table 2).

**TABLE 2:** The association of AMT score with MFQ, self-harm, suicidal thoughts and suicidal plans at age 16

AMT	*n*	Above cut point for MFQ	Self-harm	Suicidal thoughts	Suicidal plans
Observed data					
Q4 (less specific)		119 (16.6%)	122 (16.7%)	99 (13.8%)	27 (3.8%)
Q3		194 (18.4%)	214 (20.3%)	171 (16.2%)	42 (4.0%)
Q2		150 (16.1%)	175 (18.8%)	135 (14.5%)	38 (4.1%)
Q1 (more specific)		182 (18.1%)	209 (20.8%)	181 (18.0%)	56 (5.5%)
Regression models using observed data					
Model 1	3708	1.05 (0.96, 1.14), *p* = .263	1.08 (1.00, 1.17), *p* = .056	1.13 (1.04, 1.23), *p* = .006	1.18 (1.01, 1.38), *p* = .032
Model 2	3708	1.00 (0.92, 1.09), *p* = .968	1.02 (0.94, 1.11), *p* = .689	1.08 (0.98, 1.18), *p* = .104	1.14 (0.97, 1.33), *p* = .108
Model 3	3129	1.03 (0.93, 1.13), *p* = .597	1.00 (0.91, 1.10), *p* = .984	1.08 (0.97, 1.19), *p* = .145	1.21 (1.02, 1.45), *p* = .029
Model 4a	2620	0.97 (0.87, 1.09), *p* = .624	1.02 (0.92, 1.14), *p* = .652	1.05 (0.93, 1.17), *p* = .428	1.17 (0.95, 1.45), *p* = .138
Model 4b	2963	1.04 (0.94, 1.16), *p* = .423	1.00 (0.91, 1.11), *p* = .921	1.07 (0.96, 1.19), *p* = .206	1.25 (1.03, 1.52), *p* = .024
Model 4c	2454	0.98 (0.87, 1.10), *p* = .704	1.03 (0.92, 1.15), *p* = .648	1.02 (0.91, 1.15), *p* = .731	1.19 (0.94, 1.51), *p* = .137
Regression models using imputed data					
Model 1	5792	1.05 (0.97, 1.14), *p* = .220	1.09 (1.01, 1.19), *p* = .026	1.14 (1.05, 1.24), *p* = .002	1.19 (1.03, 1.38), *p* = .019
Model 2	5792	1.01 (0.93, 1.10), *p* = .739	1.03 (0.95, 1.12), *p* = .511	1.09 (1.00, 1.19), *p* = .047	1.15 (0.99, 1.33), *p* = .062
Model 3	5792	1.03 (0.95, 1.13), *p* = .448	1.04 (0.95, 1.13), *p* = .412	1.10 (1.01, 1.20), *p* = .029	1.17 (1.01, 1.37), *p* = .038
Model 4a	5792	1.00 (0.92, 1.10), *p* = .963	1.01 (0.92, 1.10), *p* = .872	1.07 (0.98, 1.17), *p* = .148	1.13 (0.97, 1.33), *p* = .107
Model 4b	5484	1.05 (0.96, 1.15), *p* = .300	1.05 (0.96, 1.15), *p* = .296	1.10 (1.00, 1.21), *p* = .057	1.16 (0.98, 1.38), *p* = .086
Model 4c	5484	1.01 (0.92, 1.11), *p* = .763	1.02 (0.93, 1.12), *p* = .700	1.05 (0.96, 1.16), *p* = .289	1.13 (0.95, 1.34), *p* = .168

Estimates shown indicate change in the odds of each binary outcome for a 1 SD change in continuous standardised AMT score.

Model 1, unadjusted effect of number of specific memories; Model 2, Model 1 adjusted for confounding effects of gender; Model 3, Model 2 further adjusted for confounding effects of SES; Model 4a, Model 3 further adjusted for depressed mood at baseline as a continuous scale; Model 4b, Model 3 re-estimated after excluding those cases above threshold for depression at baseline (“possible depression”); Model 4c, Model 3 further adjusted for depressed mood at baseline AND excluding those above threshold for depression at baseline (“possible depression”).

### AMT score and mental health outcomes

A score above the cut-off for depression on the MFQ was present for 17.4% of the sample, whilst 19.4% reported self-harm, 15.8% suicidal ideation and 4.4% suicidal planning. Despite the similar rates of self-harm and suicidal ideation, there was substantial discordance in the reporting of these two behaviours with 9.7% of the sample reporting self-harm without ideation, 6.1% ideation without self-harm and 9.7% reporting both self-harm and ideation. Unadjusted and adjusted logistic regression results examining the association between AMT performance and psychological outcomes are shown in Table 2.

##### Depression (MFQ)

There was no evidence of an association between AMT score at age 13 and depression on the MFQ at 16 years either before or after controlling for gender, SES and depressed mood at baseline. Exclusion of cases with possible depression at baseline had very little impact on results.

##### Self-harm, suicidal ideation and suicidal planning

Whilst there was moderate evidence of a negative linear relationship between AMT score and each measure in the unadjusted analysis (i.e., those with a lower AMT score—less specific memory recall—were less likely to report each outcome), these relationships were attenuated substantially on adjustment for confounders and were essentially null in the multivariable model, the only exception being suicidal planning for which there was moderate evidence following adjustment of a small protective effect of OGM, with approximately a 20% increase in the odds of suicidal planning for each one SD *increase* in memory specificity. This could not be fully explained by possible depression or depressed mood at baseline.

##### Life events and psychological outcomes

As shown in Table 3 there was strong evidence of substantial positive linear associations between the weighted life events and all four mental health outcomes (depression, self-harm, suicidal ideation and suicidal planning) which did not attenuate appreciably after controlling for gender and SES. These findings are consistent with existing research that experience of stressful life events can markedly increase risk of psychopathology in adolescence. However it should be noted that life events were reported upon retrospectively, at the same time as mental health outcomes, so it is possible that these associations in part reflect greater recollection and reporting of negative life events by those individuals who are currently depressed (mood congruent recall).

**TABLE 3:** The association between weighted life events score and MFQ, self-harm, suicidal thoughts and suicidal plans at age 16

Life events	*n*	Above cut point for MFQ	Self-harm	Suicidal thoughts	Suicidal plans
Observed data					
Q1 (fewer events)		126 (10.4%)	141 (11.6%)	104 (8.6%)	23 (1.9%)
Q2		164 (14.4%)	152 (13.3%)	120 (10.5%)	36 (3.2%)
Q3		200 (16.8%)	245 (20.6%)	209 (17.6%)	46 (3.9%)
Q4 (more events)		356 (29.7%)	355 (29.6%)	320 (26.7%)	100 (8.3%)
Regression models using observed data					
Model 1	3704	1.70 (1.57, 1.85)	1.74 (1.60, 1.88)	1.83 (1.67, 1.99)	2.00 (1.75, 2.29)
Model 2	3704	1.64 (1.51, 1.79)	1.67 (1.53, 1.81)	1.76 (1.61, 1.92)	1.94 (1.69, 2.22)
Model 3	3126	1.75 (1.59, 1.92)	1.68 (1.53, 1.85)	1.76 (1.59, 1.94)	1.90 (1.62, 2.23)
Model 4a	2617	1.67 (1.50, 1.88)	1.58 (1.41, 1.76)	1.63 (1.45, 1.83)	1.65 (1.35, 2.00)
Model 4b	2960	1.71 (1.54, 1.90)	1.65 (1.49, 1.83)	1.74 (1.57, 1.94)	1.85 (1.55, 2.21)
Model 4c	2451	1.69 (1.50, 1.91)	1.58 (1.41, 1.77)	1.66 (1.47, 1.88)	1.64 (1.30, 2.06)
Regression models using imputed data					
Model 1	5792	1.68 (1.56, 1.83)	1.77 (1.64, 1.92)	1.86 (1.71, 2.02)	2.04 (1.79, 2.33)
Model 2	5792	1.65 (1.52, 1.79)	1.71 (1.57, 1.86)	1.80 (1.65, 1.97)	2.00 (1.75, 2.28)
Model 3	5792	1.64 (1.51, 1.78)	1.72 (1.58, 1.87)	1.79 (1.64, 1.96)	1.98 (1.73, 2.27)
Model 4a	5792	1.53 (1.40, 1.66)	1.62 (1.49, 1.77)	1.66 (1.52, 1.83)	1.81 (1.57, 2.08)
Model 4b	5484	1.59 (1.46, 1.74)	1.68 (1.54, 1.84)	1.76 (1.60, 1.93)	1.92 (1.66, 2.22)
Model 4c	5484	1.53 (1.39, 1.68)	1.62 (1.48, 1.78)	1.68 (1.52, 1.86)	1.86 (1.60, 2.15)

Estimates shown indicate change in the odds of each binary outcome for a 1 SD change in standardised weighted life events scale. All *p* values <.001 are not displayed in the table.

Model 1, unadjusted effect of weighted life events; Model 2, Model 1 adjusted for confounding effects of gender; Model 3, Model 2 further adjusted for confounding effects of SES; Model 4a, Model 3 further adjusted for depressed mood at baseline as a continuous scale; Model 4b, Model 3 re-estimated after excluding those cases above threshold for depression at baseline (“possible depression”); Model 4c, Model 3 further adjusted for depressed mood at baseline AND excluding those above threshold for depression at baseline (“possible depression”).

##### Moderation of the association between life events and mental health outcomes by AMT score

Table 4 shows the results for the interaction model investigating the possible moderating effect of OGM. Here we focus on the complete case sample (*n* = 3704) prior to the adjustment for potential confounders. The main effects of life events and AMT score indicate the estimated effect of one variable when the other takes a value of zero. These effect estimates are consistent with those from Tables 2 and 3. The interaction (product) term describes the degree to which the detrimental effect of life events decreases as one’s memory specificity increases. Margins from this model are shown below as an aid to interpretation. What is apparent from these figures is that whilst the interaction effect may be in the direction hypothesised, there is no evidence in support of moderation by AMT in these data. Neither adjustment for confounders nor data imputation affected these conclusions (see Web Appendix 2; Tables X1 and X2). Further analyses showed no evidence for a differential moderating effect of AMT between males and females, i.e., a three-way interaction (details available on request).

**TABLE 4:** Testing the moderating effect of AMT score on weighted life events against psychological outcomes at age 16. Unadjusted model with complete case data (*n* = 3704)

	Above cut point for MFQ	Self-harm	Suicidal thoughts	Suicidal plans
Estimates from interaction model				
Main effect of weighted life events	1.79 (1.52, 2.10), *p* < .001	1.79 (1.52, 2.10), *p* < .001	2.01 (1.70, 2.39), *p* < .001	2.32 (1.77, 3.03), *p* < .001
Main effect of AMT	1.01 (0.97, 1.05), *p* = .580	1.02 (0.99, 1.06), *p* = .179	1.05 (1.01, 1.08), *p* = .019	1.08 (1.00, 1.16), *p* = .037
Interaction term	0.99 (0.95, 1.02), *p* = .462	0.99 (0.96, 1.03), *p* = .646	0.97 (0.94, 1.01), *p* = .165	0.96 (0.91, 1.02), *p* = .211
*Margins from interaction model to aid interpretation*				
Linear effect of weighted life events at:				
AMT = 0 (no specific memories)	1.79 (1.52, 2.10)	1.79 (1.52, 2.10)	2.01 (1.70, 2.39)	2.32 (1.77, 3.03)
AMT = 2	1.75 (1.57, 1.93)	1.77 (1.58, 1.95)	1.92 (1.72, 2.16)	2.16 (1.79, 2.59)
AMT = 4	1.70 (1.57, 1.84)	1.73 (1.60, 1.88)	1.82 (1.67, 1.99)	2.01 (1.75, 2.29)
AMT = 6	1.65 (1.49, 1.84)	1.70 (1.54, 1.90)	1.73 (1.55, 1.93)	1.86 (1.58, 2.20)
AMT = 8	1.62 (1.38, 1.90)	1.68 (1.43, 1.95)	1.65 (1.40, 1.93)	1.73 (1.35, 2.23)
AMT = 10 (all specific memories)	1.57 (1.26, 1.95)	1.65 (1.34, 2.05)	1.57 (1.25, 1.97)	1.62 (1.14, 2.29)

All parameter estimates are odds ratios with 95% confidence intervals. Life events in standardised such that estimates indicate change on odds for a 1 SD increase in weighted life events. For AMT, estimates indicate change in odds for an increase of one specific memory.

## DISCUSSION

There has been considerable interest in the suggestion that OGM may be a vulnerability marker for the initial development of major depression and previous research has suggested that OGM moderates the association between negative life events and depressive symptoms in adolescent and young adult samples. However a majority of these studies have been based on high-risk samples, and most have considered only relatively short periods of follow-up. Further, none have examined the association between OGM and either self-harm or suicidal ideation. Our results showed no evidence of associations between OGM at age 13 and depressive symptoms at age 16 in this low risk, community sample. Although unadjusted analyses indicated direct negative associations between OGM and self-harm, suicidal ideation and suicidal planning, in the former two cases these were attenuated after adjustment for gender (which was associated with both less OGM and more depression). In the latter case there was weak evidence of an association for suicidal planning that was not adequately explained by adjustment for SES. This is consistent with work that suggests that OGM is associated with a more general lack of specificity in forming goals and plans (Crane, Winder, Hargus, Amarasinghe, & Barnhofer, 2012) and the findings that in non-clinical samples OGM can have protective effects (e.g., Hermans et al., 2008; Raes et al., 2006). However the large number of comparisons computed means that this result should be interpreted with caution. There was no evidence of a moderating effect of OGM on the association between life events and mental health outcomes.

The findings of the current study are broadly consistent with the conclusions of Sumner et al. (2010), that OGM may be more powerful as a short-term than long-term predictor of depressive symptoms. The current study utilised a long follow-up period and it is possible either that the impact of life events on psychopathology is attenuated over this longer time interval and/or that OGM is simply not sufficiently stable to act as a predictor of psychopathology nearly three years later, particularly across a period of rapid development in adolescence. Sumner et al.’s research has further suggested that there is no association between OGM when assessed outside depressive episodes and later depression symptoms, and the results of this community study are consistent with this conclusion. As Raes et al. (2006) have pointed out, OGM may first emerge during cognitive development as an appropriate mood-repair mechanism to moderate emotional reactions to stressful events. Only in some people—those who undergo sustained stress or adversity—does over generality become an inflexible pattern of responding that reduces problem-solving ability and increases risk of future persistent depression or PTSD (Williams et al. 2007). Once depression has occurred in the life course of an individual, further stress or low mood can reactivate a pattern of reaction (e.g., rumination) that itself makes further depression more likely, and it is here that OGM seems to add further vulnerability, combining with rumination to make low mood persist (Hamlet et al., 2014; see also Brennan, Barnhofer, Crane, Duggan, & Williams, in press; Owens et al., 2014).

We do not yet know which of the contributory mechanisms underlying OGM may play a role in this developmental sequence. Research suggests that over-general responding on the AMT is influenced by a number of different processes, including impaired executive functioning, a ruminative cognitive style and avoidance tendencies (e.g., Sumner et al., 2011; Williams et al., 2007) and that the extent to which individual differences in OGM index each of these processes differs as a function of the population under investigation, and complex interactions between different CaR–FA–X mechanism are emerging (Sumner et al., 2014). Furthermore, research has shown that, in depressed populations, executive function impairments seem to contribute more strongly to OGM whereas in patients with PTSD, avoidance plays a bigger role (e.g., Dalgleish, Rolfe, Golden, Dunn, & Barnard, 2008; Dalgleish et al., 2007). Impaired executive functioning, rumination and avoidance tendencies themselves confer risk of poor mental health and it is possible that individual differences in OGM are associated with poor mental health outcomes only to the extent that they reflect impaired executive functioning, rumination or avoidance processes. Individual differences in OGM in community samples may reflect other factors (such as motivation or disclosure tendencies) that are less closely associated with vulnerability to later depression. It is possible that individual differences in OGM in high-risk samples are more akin to those observed in individuals with a pre-existing depressive illness, but that individual differences in community samples have little implication for vulnerability to psychopathology. Indeed the absence of any associations between OGM and mental health outcomes in this sample supports the conclusion that OGM is not a marker of vulnerability to psychopathology in community samples, though this conclusion has to be tempered with the possibility that the base rate of OGM in this community sample might have been too low to detect an effect.

### Limitations

There are a number of aspects of the study design that should be considered when interpreting the results. First, due to the large scale of the study, a written self-completed version of the AMT was used. Although written assessment of memory specificity has been validated in previous research (e.g., Raes et al., 2006, 2010) it is nevertheless likely to have reduced the sensitivity with which OGM was assessed and increased the extent to which performance on the task reflected motivational factors, relative to administration under strictly controlled conditions. It is therefore possible that stronger associations would have been observed between OGM and later mental health had individual face-to-face testing been conducted. Furthermore, AMT was only tested on one occasion, so may not be as reliable an indicator of over generality as might have been achievable by repeated testing, and also prevents us from knowing what impact any change in OGM over time might have had. Note that using a questionnaire measure also means that we cannot be sure why participants failed to respond to some of the items. There is the danger that the results are limited because we are not assessing OGM, but rather fatigue or lack of interest in completing the questionnaire. However, in our earlier article describing the autobiographical memory data from this cohort in detail (Heron et al., 2012), we were able to examine the nature of the omissions and found that there was no greater probability of participants omitting the later items than the earlier, suggesting that they were not simply getting tired or losing interest.

Second, the measure of life events taken at age 16 used a checklist of events rather than an interview. Although this was necessitated by the size of the cohort, we acknowledge that contextualised information is lost by this procedure (Liu, 2013). Another limitation is that we did not attempt to categorise life events along dimensions such as “interpersonal” versus “achievement”, and future studies should examine this question explicitly (see Stange, Hamilton, Abramson, & Alloy, 2014). Furthermore, the life events checklist referred to events experienced since 12 years of age. As a result it is possible that some of the life events reported may have preceded the measurement of AMT testing. However since no interaction was identified between OGM and life events this limitation has less relevance.

Third, the measure of self-harm used was a lifetime measure (“Have you ever …”) rather than an assessment of behaviour taking place between 12 and 16 years of age. However, suicidal behaviour before the age of 12 is extremely rare, so we do not consider that our analysis of OGM as a moderator of the risk for “future” self-harm is unduly compromised.

Fourth, we did not assess other possible variables that could shed light on what factors might combine with OGM to moderate the impact of life events on depression. For example, Hamlat et al. (2014) found that, in a community sample of boys and girls aged 12–13, depression in girls was more likely nine months later if they had shown more OGM and a greater tendency to ruminate. Future research should ensure that all aspects of the CaR–FA–X model are tested in community samples.

Fifth, the group of individuals reporting self-harm was comprised of those harming with and those harming without suicidal ideation. Indeed many respondents may have exhibited both behaviours, and it is possible that any effect may be being driven by the subgroup who had harmed with intent. However, as this subgroup is likely to overlap strongly with those reporting suicidal thoughts or plans we do not feel our decision is likely to be masking any noteworthy findings.

Finally, the measure of depression at age 16 referred to current symptoms only and adolescents who had experienced isolated depressive episodes earlier in their life would not have been identified. Again the association between OGM and later mental health may have been underestimated as a result.

However, despite these limitations the study also has important strengths. It is the largest study to date to explore associations between OGM and later adolescent psychopathology in a community sample and one of the few that has been able to adequately adjust for sociodemographic confounders. The effects of gender, SES and depressed mood on OGM and the impact of adjustment for these factors on the observed associations suggest that it is likely to be critical to consider and control for these factors in future studies.

### Conclusions

The findings of the current study suggest that at least in community samples, OGM assessed in early adolescence is not related to mid-adolescent psychopathology, either alone, or in interaction with experience of stressful life events. The findings are consistent with accumulating evidence that increased memory over generality, when assessed in individuals without current symptoms of depression or other significant risk factors, does not usually relate to subsequent risk of naturally occurring depressive symptoms. Although there were some associations identified between OGM and variables indexing reduced presence of suicidal planning, these need to be seen in the light of the large number of comparisons computed and the overall pattern of results which suggested a lack of association between age 13 OGM and mental health outcomes. Further research is therefore required in order to clarify whether there is any reliable association between OGM and various manifestations of suicidal ideation and self-harm in adolescence. On the basis of this accumulating evidence we argue that while individual differences in memory specificity may remain a vulnerability marker in high risk or clinic samples, it should not be regarded as a marker of vulnerability to adolescent psychopathology in general community samples.

## Supplementary Material

Supplementary_Material.docx
