# Supplementary material for: Adolescent over-general memory, life events and mental health outcomes: Findings from a UK cohort study
Source: Memory. 2015 Feb 26;24(3):348–63. doi: 10.1080/09658211.2015.1008014 (PMC4743605; doi:10.1080/09658211.2015.1008014)
Supplement: Supplementary_Material.docx [file pmem_a_1008014_sm8839.docx]

**Web Appendix 1. Additional data showing social patterning of AMT and weighted life events**

Web Table 1. *The association between demographic variables and autobiographical memory test score (quartiles):*

|  |  | Quartiles of AMT | | | |  |
| --- | --- | --- | --- | --- | --- | --- |
|  |  | Q1 (best) | Q2 | Q3 | Q4 (worst) | X2, p |
| Gender | Male | 539 (36.4%) | 527 (37.9%) | 751 (45.8%) | 678 (52.9%) | X2 = 97.2 p < .001 |
|  | Female | 942 (63.6%) | 862 (62.1%) | 889 (54.2%) | 604 (47.1%) |  |
| Housing tenure | Mortgaged/owned | 1217 (84.6%) | 1164 (86.3%) | 1313 (82.4%) | 998 (80.2%) | X^2^ = 40.1 p < .001 |
|  | Private rented | 129 (9.0%) | 104 (7.7%) | 119 (7.5%) | 105 (8.4%) |  |
|  | Subsidized rented | 93 (6.5%) | 81 (6.0%) | 161 (10.1%) | 142 (11.4%) |  |
| Parity | First born | 709 (49.1%) | 644 (48.0%) | 787 (49.3%) | 561 (45.5%) | X^2^ = 14.7 p = .022 |
|  | Second born | 494 (34.2%) | 503 (37.5%) | 541 (33.9%) | 432 (35.1%) |  |
|  | Third born plus | 240 (16.6%) | 195 (14.5%) | 268 (16.8%) | 239 (19.4%) |  |
| Home overcrowding | ≤1 person/room | 1390 (97.2%) | 1292 (96.6%) | 1517 (95.8%) | 1157 (94.7%) | X^2^ = 12.6 p = .006 |
|  | >1 person/room | 40 (2.8%) | 45 (3.4%) | 66 (4.2%) | 65 (5.3%) |  |
| Maternal education | A level or higher | 759 (52.6%) | 643 (47.4%) | 650 (40.9%) | 434 (35.2%) | X^2^ = 108.7 p < .001 |
|  | O-level | 457 (31.7%) | 469 (34.6%) | 581 (36.5%) | 470 (38.1%) |  |
|  | < O-level | 227 (15.7%) | 244 (18.0%) | 360 (22.6%) | 330 (26.7%) |  |
| Household income | Top 20% | 364 (27.3%) | 336 (26.8%) | 297 (20.0%) | 214 (18.9%) | X^2^ = 61.1 p < .001 |
|  | Middle 60% | 819 (61.4%) | 758 (60.4%) | 937 (63.1%) | 711 (62.7%) |  |
|  | Lowest 20% | 152 (11.4%) | 161 (12.8%) | 250 (16.9%) | 209 (18.4%) |  |
| Social class | Professional/managerial & technical | 964 (69.6%) | 850 (65.1%) | 911 (59.9%) | 643 (55.2%) | X^2^ = 63.9 p < .001 |
|  | Skilled non-manual or lower | 422 (30.5%) | 455 (34.9%) | 610 (40.1%) | 521 (44.8%) |  |
| Marital status at enrolment | Married (incl. divorced/widowed) | 1270 (87.4%) | 1191 (87.6%) | 1377 (85.5%) | 1050 (84.3%) | X^2^ = 8.3 p = .040 |
|  | Unmarried | 183 (12.6%) | 169 (12.4%) | 234 (14.5%) | 195 (15.7%) |  |
| Maternal age at delivery | < 25 years | 192 (13.0%) | 178 (12.8%) | 269 (16.4%) | 238 (18.6%) | X^2^ = 34.9 p < .001 |
|  | 25-29 | 544 (36.7%) | 553 (39.8%) | 645 (39.3%) | 496 38.7%) |  |
|  | 30-34 | 543 (36.7%) | 485 (34.9%) | 528 (32.2%) | 412 (32.1%) |  |
|  | 35+ | 173 (13.6%) | 198 (12.5%) | 136 (12.1%) | 709 (10.6%) |  |

Web Table 2. *The association between demographic variables and weighted life events score at age 16.*

|  |  | Quartiles of weighted life events | | | |  |
| --- | --- | --- | --- | --- | --- | --- |
|  |  | Q1 (lowest) | Q2 | Q3 | Q4 (highest) | X2, p |
| Gender | Male | 592 (47.2%) | 530 (44.7%) | 472 (38.4%) | 412 (33.7%) | X^2^=57.2,  p < .001 |
|  | Female | 663 (52.8%) | 655 (55.3%) | 756 (61.6%) | 812 (66.3%) |  |
| Housing tenure | Mortgaged/owned | 1087 (89.4%) | 997 (86.3%) | 1012 (84.5%) | 941 (79.8%) | X^2^=50.9,  p < .001 |
|  | Private rented | 69 (5.7%) | 91 (7.9%) | 106 (8.9%) | 114 (9.7%) |  |
|  | Subsidized rented | 60 (4.9%) | 68 (5.9%) | 79 (6.6%) | 124 (10.5%) |  |
| Parity | First born | 665 (54.4%) | 608 (52.8%) | 543 (45.2%) | 511 (43.0%) | X^2^=69.0,  p < .001 |
|  | Second born | 414 (33.9%) | 383 (33.3%) | 451 (37.6%) | 418 (35.2%) |  |
|  | Third born plus | 144 (11.8%) | 160 (13.9%) | 207 (17.2%) | 259 (21.8%) |  |
| Home overcrowding | ≤1 person/room | 1173 (97.3%) | 1114 (97.2%) | 1146 (96.6%) | 1114 (95.4%) | X^2^=8.7,  p < .001 |
|  | >1 person/room | 32 (2.7%) | 32 (2.8%) | 41 (3.5%) | 54 (4.6%) |  |
| Maternal education | A level or higher | 567 (46.2%) | 575 (49.4%) | 620 (52.0%) | 529 (44.8%) | X^2^=20.1,  p < .001 |
|  | O-level | 432 (35.2%) | 388 (33.4%) | 370 (31.0%) | 395 (33.5%) |  |
|  | < O-level | 228 (18.6%) | 200 (17.2%) | 203 (17.0%) | 257 (21.8%) |  |
| Household income | Top 20% | 306 (26.8%) | 301 (28.0%) | 291 (25.8%) | 253 (22.9%) | X^2^=22.6,  p < .001 |
|  | Middle 60% | 699 (61.3%) | 643 (59.9%) | 677 (59.9%) | 660 (59.7%) |  |
|  | Lowest 20% | 135 (11.8%) | 130 (12.1%) | 162 (14.3%) | 193 (17.5%) |  |
| Social class | Professional/managerial & technical | 748 (63.4%) | 738 (66.5%) | 752 (66.0%) | 721 (63.4%) | X^2^=4.0,  p = .300 |
|  | Skilled non-manual or lower | 431 (36.6%) | 372 (33.5%) | 387 (34.0%) | 416 (36.6%) |  |
| Marital status at enrolment | Married (incl. divorced/widowed) | 1098 (88.8%) | 1024 (87.7%) | 1052 (87.6%) | 1009 (85.0%) | X^2^=8.5,  p < .001 |
|  | Unmarried | 138 (11.2%) | 144 (12.3%) | 149 (12.4%) | 178 (15.0%) |  |
| Maternal age at delivery | < 25 years | 172 (13.7%) | 156 (13.2%) | 165 (13.4%) | 195 (15.9%) | X^2^=24.2, p = .004 |
|  | 25-29 | 511 (40.7%) | 470 (39.7%) | 454 (37.0%) | 416 (34.0%) |  |
|  | 30-34 | 419 (33.4%) | 424 (35.8%) | 423 (34.5%) | 429 (35.1%) |  |
|  | 35+ | 153 (12.2%) | 135 (11.4%) | 186 (15.2%) | 184 (15.0%) |  |

**Web Appendix 2. Further information relating to interaction model for moderation by AMT**

We anticipated that the detrimental effect of life events should be more marked for respondents with over-general memory as illustrated below:-

| Life events  Log-odds of outcome  (e.g. self-harm)  Increasingly  over-general  memory |
| --- |

We tested this using an interaction model containing:-

- AMT (number of specific responses)
- Life events (weighted by severity, and standardized to aid interpretation)
- The interaction (product) of AMT and standardized life events

Given the direction of the variables of interest, an interaction term of negative sign (or below one on the odds scale) would be consistent with the above figure – namely, that the effect of life events diminishes with increasingly specific memory recall. To aid interpretation, the -margins- procedure in Stata was used to illustrate the effect of one variable across levels of the other. Note these are model-based estimates rather than stratum specific effects so they preserve the continuous nature of the data. Finally note, preliminary univariable analyses were in support of linear effects for these data so we carry this forward into these interaction models.

Estimates for the unadjusted model using the complete case dataset are shown in table X1 overleaf. Given the parameterization of the constituent variables:-

1. The main effect of life events is estimated at the point AMT = zero, i.e. no specific memories. The strong positive association of life events is consistent with the estimates in Manuscript table 3.
2. The main effect of AMT is estimated at the mean of life events. This is mildly positive, indicating a subtle increase in risk for those with more specific memory recall – consistent with the results from table 2.
3. Finally, the interaction term is below one (consistent with the figure above)

The margins are shown for completeness despite the lack of evidence for an interaction. They illustrate a slight decrease in the detrimental effect of life events as the number of specific memories increases. Whilst our estimates are in the direction of our theorized relationship, the evidence (as indicated by the interaction term) is insufficient for us to conclude that moderation by AMT is present.

Table X1. The moderating effect of autobiographical memory test score on weighted life events against psychological outcomes at age 16.

|  | Above cut-point for MFQ | Self-harm | Suicidal thoughts | Suicidal plans |
| --- | --- | --- | --- | --- |
| *Estimates from interaction model* | |  |  |  |
| Main effect of life events | 1.79 [1.52, 2.10] p < .001 | 1.79 [1.52, 2.10] p < .001 | 2.01 [1.70, 2.39] p < .001 | 2.32 [1.77, 3.03] p < .001 |
| Main effect of AMT | 1.01 [0.97, 1.05] p = .580 | 1.02 [0.99, 1.06] p = .179 | 1.05 [1.01, 1.08] p =0.019 | 1.08 [1.00, 1.16] p =.037 |
| Interaction term | 0.99 [0.95, 1.02] p = .462 | 0.99 [0.96, 1.03] p = .646 | 0.97 [0.94, 1.01] p = .165 | 0.96 [0.91, 1.02] p = .211 |
|  |  |  |  |  |
| *Margins from interaction model to assist with interpretation* | | |  |  |
| Linear effect of life events at: |  |  |  |  |
| AMT = 0 | 1.79 [1.52, 2.10] | 1.79 [1.52, 2.10] | 2.01 [1.70, 2.39] | 2.32 [1.77, 3.03] |
| AMT = 2 | 1.75 [1.57, 1.93] | 1.77 [1.58, 1.95] | 1.92 [1.72, 2.16] | 2.16 [1.79, 2.59] |
| AMT = 4 | 1.70 [1.57, 1.84] | 1.73 [1.60, 1.88] | 1.82 [1.67, 1.99] | 2.01 [1.75, 2.29] |
| AMT = 6 | 1.65 [1.49, 1.84] | 1.70 [1.54, 1.90] | 1.73 [1.55, 1.93] | 1.86 [1.58, 2.20] |
| AMT = 8 | 1.62 [1.38, 1.90] | 1.68 [1.43, 1.95] | 1.65 [1.40, 1.93] | 1.73 [1.35, 2.23] |
| AMT = 10 | 1.57 [1.26, 1.95] | 1.65 [1.34, 2.05] | 1.57 [1.25, 1.97] | 1.62 [1.14, 2.29] |

Unadjusted model (model 1) with complete case data (n = 3,704) All parameter estimates are Odds Ratios with 95% CI. Life events in standardized such that estimates indicate change on odds for a one SD increase in weighted life events. For AMT, estimates indicate change in odds for an increase of one specific memory.

Table X2 overleaf shows a complete set of estimated interaction effects for the complete case samples and imputed data. The conclusion that there is an absence of moderation by AMT is upheld throughout. We also note that the parameter estimates for the interaction effect are less variable when the missing data is dealt with. This phenomenon was apparent for many of the models carried out for this manuscript. When focussing on complete case samples, the drop in sample size often led to an increase in the evidence against the null hypothesis. This does not occur when the sample size is stable.

Table X2. Full set of estimated interaction effects

|  | n | Above cut-point for MFQ | Self-harm | Suicidal thoughts | Suicidal plans |
| --- | --- | --- | --- | --- | --- |
| *Complete case models* | | |  |  |  |
| Model 1 | 3,704 | 0.99 [0.95, 1.02] p = .462 | 0.99 [0.96, 1.03] p = .646 | 0.97 [0.94, 1.01] p = .165 | 0.96 [0.91, 1.02] p = .211 |
| Model 2 | 3,704 | 0.99 [0.96, 1.03] p = .582 | 1.00 [0.96, 1.03] p = .852 | 0.98 [0.94, 1.01] p = .231 | 0.97 [0.91, 1.02] p = .250 |
| Model 3 | 3,126 | 0.97 [0.93, 1.01] p = .090 | 0.98 [0.94, 1.02] p = .280 | 0.98 [0.94, 1.02] p = .321 | 0.97 [0.91, 1.04] p = .437 |
| Model 4a | 2,617 | 0.97 [0.93, 1.02] p = .288 | 1.00 [0.96, 1.05] p = .960 | 0.98 [0.93, 1.03] p = .421 | 0.98 [0.90, 1.06] p = .597 |
| Model 4b | 2,960 | 0.98 [0.94, 1.02] p =0.315 | 0.98 [0.94, 1.02] p = .365 | 0.99 [0.95, 1.04] p = .705 | 0.99 [0.92, 1.06] p = .758 |
| Model 4c | 2,451 | 0.99 [0.94, 1.04] p = .579 | 1.00 [0.96, 1.05] p = .939 | 0.99 [0.94, 1.04] p = .695 | 0.99 [0.91, 1.09] p = .906 |
| *Models following missing data imputation* | | |  |  |  |
| Model 1 | 5,792 | 0.99 [0.96, 1.02] p = .513 | 0.99 [0.96, 1.03] p = .775 | 0.98 [0.94, 1.01] p = .196 | 0.96 [0.91, 1.02] p = .158 |
| Model 2 | 5,792 | 0.99 [0.96, 1.02] p = .556 | 1.00 [0.96, 1.03] p = .863 | 0.98 [0.94, 1.01] p = .223 | 0.96 [0.91, 1.02] p = .168 |
| Model 3 | 5,792 | 0.99 [0.96, 1.02] p = .455 | 1.00 [0.96, 1.03] p = .832 | 0.98 [0.94, 1.01] p = .213 | 0.96 [0.91, 1.01] p = .140 |
| Model 4a | 5,792 | 0.99 [0.96, 1.02] p = .608 | 1.00 [0.96, 1.04] p = .983 | 0.98 [0.95, 1.02] p = .316 | 0.96 [0.91, 1.02] p = .188 |
| Model 4b | 5,484 | 1.00 [0.96, 1.03] p = .788 | 0.99 [0.96, 1.03] p = .742 | 0.98 [0.95, 1.02] p = .366 | 0.96 [0.91, 1.01] p = .145 |
| Model 4c | 5,484 | 1.00 [0.96, 1.03] p = .815 | 0.99 [0.96, 1.03] p = .763 | 0.98 [0.95, 1.02] p = .38 | 0.96 [0.91, 1.01] p = .141 |

Model 1: Unadjusted effect of weighted life event scale, used as a continuous standardized measure

Model 2: Model 1 adjusted for confounding effects of gender

Model 3: Model 2 further adjusted for confounding effects of SES

Model 4a: Model 3 further adjusted for depressed mood at baseline as a continuous scale

Model 4b: Model 3 re-estimated after excluding those cases above threshold for depression at baseline (“possible depression”)

Model 4c: Model 3 further adjusted for depressed mood at baseline AND excluding those above threshold for depression at baseline (“possible depression”)
